# Expert Consensus Practice Recommendations of the North American Neuroendocrine Tumor Society for the management of high grade gastroenteropancreatic and gynecologic neuroendocrine neoplasms

**DOI:** 10.1530/ERC-22-0206

**Published:** 2023-07-11

**Authors:** Jennifer R Eads, Thorvardur R Halfdanarson, Tim Asmis, Andrew M Bellizzi, Emily K Bergsland, Arvind Dasari, Ghassan El-Haddad, Michael Frumovitz, Joshua Meyer, Erik Mittra, Sten Myrehaug, Eric Nakakura, Nitya Raj, Heloisa P Soares, Brian Untch, Namrata Vijayvergia, Jennifer A Chan

**Affiliations:** 1 Division of Hematology and Oncology, Abramson Cancer Center, University of Pennsylvania, Pennsylvania, USA; 2 Division of Medical Oncology, Mayo Clinic Cancer Center, Rochester, Minnesota, USA; 3 Division of Medical Oncology, University of Ottawa, Ottawa, Ontario, Canada; 4 Department of Pathology, University of Iowa Carver College of Medicine, Iowa City, Iowa, USA; 5 Department of Medicine, University of California, San Francisco, California, USA; 6 Division of Gastrointestinal Oncology, The University of Texas MD Anderson Cancer Center, Houston, Texas, USA; 7 Department of Diagnostic Imaging and Interventional Radiology, Moffitt Cancer Center and Research Institute, Tampa, Florida, USA; 8 Division of Gynecologic Oncology, The University of Texas MD Anderson Cancer Center, Houston, Texas, USA; 9 Department of Radiation Oncology, Fox Chase Cancer Center, Philadelphia, Pennsylvania, USA; 10 Division of Molecular Imaging and Therapy, Oregon Health & Science University, Portland, Oregon, USA; 11 Department of Radiation Oncology, Odette Cancer Centre, Sunnybrook Health Sciences Centre, Toronto, Ontario, Canada; 12 Department of Surgery, University of California, San Francisco, California, USA; 13 Department of Medicine, Gastrointestinal Oncology Service, Memorial Sloan Kettering Cancer Center, New York, New York, USA; 14 Division of Oncology, Huntsman Cancer Institute, University of Utah, Salt Lake City, Salt Lake City, Utah, USA; 15 Department of Surgery, Memorial Sloan Kettering Cancer Center, New York, New York, USA; 16 Department of Hematology and Oncology, Fox Chase Cancer Center, Philadelphia, Pennsylvania, USA; 17 Department of Medical Oncology, Dana-Farber Cancer Institute, Boston, Massachusetts, USA

**Keywords:** neuroendocrine tumors, neuroendocrine carcinoma, recommendations, high-grade

## Abstract

High-grade neuroendocrine neoplasms are a rare disease entity and account for approximately 10% of all neuroendocrine neoplasms. Because of their rarity, there is an overall lack of prospectively collected data available to advise practitioners as to how best to manage these patients. As a result, best practices are largely based on expert opinion. Recently, a distinction was made between well-differentiated high-grade (G3) neuroendocrine tumors and poorly differentiated neuroendocrine carcinomas, and with this, pathologic details, appropriate imaging practices and treatment have become more complex. In an effort to provide practitioners with the best guidance for the management of patients with high-grade neuroendocrine neoplasms of the gastrointestinal tract, pancreas, and gynecologic system, the North American Neuroendocrine Tumor Society convened a panel of experts to develop a set of recommendations and a treatment algorithm that may be used by practitioners for the care of these patients. Here, we provide consensus recommendations from the panel on pathology, imaging practices, management of localized disease, management of metastatic disease and surveillance and draw key distinctions as to the approach that should be utilized in patients with well-differentiated G3 neuroendocrine tumors vs poorly differentiated neuroendocrine carcinomas.

## Introduction

High-grade neuroendocrine neoplasms (NENs) constitute a rare disease entity and account for approximately 10% of all NENs. Given their rarity, there is a paucity of prospective data to guide the optimal diagnosis and management of these patients. In recent years, updates to the pathologic classification of high-grade NENs have made this additionally complex with high-grade NENs now being subcategorized based on tumor differentiation. These subcategories include well-differentiated high-grade neuroendocrine tumors (G3 NET) and poorly differentiated high-grade neuroendocrine carcinomas (NECs). Most available literature does not account for this subcategorization and as a result, much of the management of high-grade NENs is based on expert opinion. Extra-pulmonary G3 NETs most commonly arise within the pancreas, followed by the small bowel. NECs most commonly arise within the lower gastrointestinal tract (colon and rectum), upper gastrointestinal tract (stomach and esophagus), and pancreas (Liu *et al.* 2021). Other commonly reported NEC sites of origin are in the genitourinary tract and the female pelvic organs but up to one-third of NECs are of the unknown primary site (Dasari *et al.* 2018). The prognosis of patients with metastatic G3 NET is fair, averaging around 42 months (Liu *et al.* 2021). Unfortunately, NEC has a much poorer prognosis with the median overall survival (OS) being less than a year, particularly for primaries arising from sites other than the small bowel and appendix (Dasari *et al.* 2017, 2018, 2022). In an effort to provide guidance on best treatment practices, we now put forth these consensus practice recommendations from the North American Neuroendocrine Tumor Society (NANETS) for the management of high-grade NENs of gastroenteropancreatic and gynecologic origin.

## Methods for developing consensus practice recommendations

**Figure 1:**
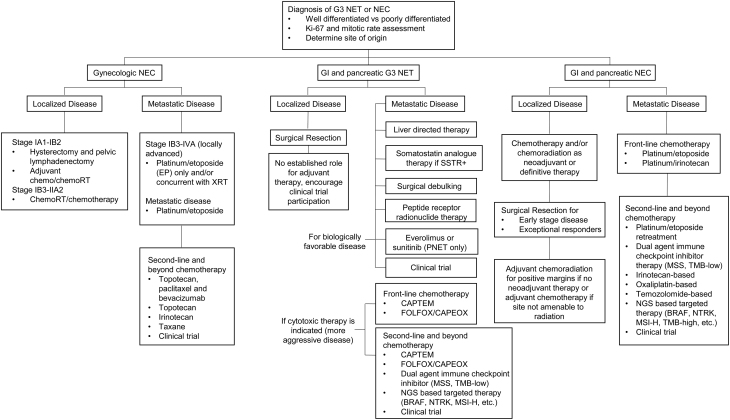
Management of high-grade neuroendocrine neoplasms.

A panel consisting of 17 neuroendocrine experts from 13 institutions was convened by the NANETS Publications and Guidelines Committee. This panel consisted of nine medical oncologists, two radiation oncologists, two nuclear medicine physicians, one of whom is also an interventional radiologist, two surgeons, one gynecologic oncologist, and one pathologist. A subgroup of the panelists identified key topics for inclusion in these recommendations, all of which were vetted by the whole panel. Topics were assigned to appropriate panel members to conduct an extensive literature search and a face-to-face consensus meeting was held from October 2nd to 3rd, 2019, to discuss those findings. Surveys were conducted to ascertain consensus vs majority vs lack of consensus on multiple topics, generally where data was less clear. Panelists were permitted to abstain from voting on a particular topic if they did not feel they held the appropriate expertise. Consensus was defined as no more than one individual disagreeing with the rest of the voting group, majority as at least 75% of voting participants agreeing on a topic and lack of consensus as less than 75% of voting participants agreeing on a topic. Additional data to supplement the initial literature search were identified during the course of manuscript writing into 2022. Detailed recommendations are outlined later and are summarized in Fig. 1. All panelists contributed to the writing of these recommendations.

## Initial assessment

### Pathology

#### Definition and WHO criteria

High-grade NENs include G3 NET and NEC. The *World Health Organization Classification of Tumours* series of organ-system-based ‘Blue Books’ represents the gold standard for tumor classification. The diagnostic entity of G3 NET was first recognized in the pancreas and codified in the 2017 *Endocrine Tumours Blue Book* and then extended to encompass all gastroenteropancreatic sites in the 2019 *Digestive Tumours Blue Book* (World Health Organization 2019). G3 NETs are well-differentiated while NECs are poorly differentiated and include small-cell and large-cell variants. The former diagnosis is made predominantly based on morphology, while the latter formally requires the demonstration of general neuroendocrine marker expression to distinguish it from morphologic mimics. Diagnostic criteria for G3 NENs require a Ki-67 proliferation index > 20% and/or mitotic count > 20 per 2 mm^2^ though the vast majority of NECs well exceed these thresholds (Travis *et al.* 2015, Rinde *et al.* 2022).

#### Histology

Small-cell NEC is usually readily recognizable on hematoxylin-and-eosin stained slides, characterized by a high nucleus:cytoplasmic ratio, typically fusiform nuclei, finely granular chromatin, and frequent necrosis. Large-cell NEC often demonstrates organoid architecture (e.g. nested, trabecular); nuclei tend to be round with prominent nucleoli, and cytoplasm is fairly abundant; necrosis is, again, often prominent. NECs, particularly those of extra-pulmonary viscera, may arise in association with a non-NEC (in the digestive system, such tumors are referred to as mixed neuroendocrine-nonneuroendocrine neoplasm). G3 NET may demonstrate either typical well-differentiated histomorphology (including organoid architecture, low nucleus:cytoplasm ratio, finely granular chromatin) or show ambiguous histomorphology that is indeterminate for G3 NET vs large-cell NEC. Immunohistochemistry (IHC) is useful in these latter cases.

#### Immunohistochemistry

IHC is used to confirm a high-grade NEN’s neuroendocrine epithelial nature, for grading purposes (i.e. Ki-67), and to determine the site of origin in metastases of unknown primary. NENs are nearly always positive for broad-spectrum low molecular weight or pankeratins (e.g. CAM5.2, AE1/AE3); antibodies to EMA and/or EpCAM can be used in rare negative cases. NETs are nearly always positive for synaptophysin, though several diagnostic mimics may also be positive. As such, chromogranin A is typically performed concurrently. In contrast, up to one-quarter of NECs are negative for all traditional general neuroendocrine markers. INSM1 has emerged as the most sensitive general neuroendocrine marker in this setting, expressed by up to 95% of NECs (Rooper *et al.* 2017).

Ki-67 IHC is mandatory for the grading of extra-pulmonary NET, and in the vast majority of cases, the diagnosis is made based on a proliferation index > 20%, rather than a mitotic count > 20 per 2 mm^2^. Ki-67 IHC may be useful in NEC, as it is predictive and prognostic. Studies have identified various Ki-67 cutpoints in regard to prognosis (Sorbye *et al.* 2013, Walter *et al.* 2017) but there is a lack of consensus as to if any specific Ki-67 cut point should be used as it generally does not affect the treatment approach. Though the *Digestive Tumours Blue Book* recommends evaluating the Ki-67 proliferation index in ‘hot-spots’ encompassing at least 500 cells, proliferation indices in NEC are typically uniformly very high. In contrast, in many G3 NETs, foci of high proliferation are more limited in extent. Although the median proliferation index in NEC (80% in one recent series) exceeds that in G3 NET (50% in the same series), the ranges are overlapping such that Ki-67 should not be used alone in the distinction of these two tumor types (Tang* et al.* 2016*a*). Additional useful markers in this diagnostic setting include p53 (missense-mutation and null-pattern staining support a diagnosis of NEC), Rb (loss of staining supports a diagnosis of NEC), SSTR2A (diffuse, strong staining favors a diagnosis of NET, though up to one-third of NECs demonstrate strong staining), and CXCR4 (diffuse, strong staining favors a diagnosis of NEC, though this marker is not widely available).

Site of origin assignment in metastatic NEC of the unknown primary has historically been only of academic interest, as platinum and etoposide are standard therapy for distant disease independent of the primary site. With first-line immunotherapy having emerged as the standard of care in advanced Merkel cell carcinoma and with extra-pulmonary visceral NECs being increasingly treated with organ-specific regimens typically used in non-NECs, site of origin assignment is increasingly important. Despite this, extensive IHC testing to identify the primary site of origin is not performed by most pathologists. Transcription factor IHC can facilitate NET site of origin assignment, with most occult primaries arising in the midgut (CDX2) or pancreas (islet 1, PAX6 or polyclonal PAX8, PR), and fewer cases arising in the lung (OTP, TTF-1) or rectum (SATB2).

#### G3 NET vs NEC – molecular considerations

While G3 NET and NEC are often readily distinguished by morphology, the diagnosis may be challenging, especially in small biopsies. The presence of a lower grade (i.e. G1/2) NET component confirms the diagnosis of G3 NET, while a concurrent non-NEC component strongly supports a diagnosis of NEC. NEC outnumbers G3 NET on the order of 90:1 (Bellizzi 2020*a*). The molecular genetic hallmark of small-cell lung cancer is biallelic inactivation of the *TP53* and *RB1* tumor suppressors (George *et al.* 2015). This is also the case in many large-cell NECs and extra-pulmonary visceral NECs, but mutations are less frequent than in small-cell lung cancer (Sorbye *et al.* 2014, Bergsland *et al.* 2016, George *et al.* 2018) and vary considerably depending on the site of origin (Uccella *et al.* 2021, Venizelos *et al.* 2021, Metovic *et al.* 2022, Yachida *et al.* 2022). The IHC distinction of NEC from G3 NET largely relies on protein correlates of these molecular genetic events, with mutant-pattern p53 staining (either missense mutation- or null-pattern) and/or Rb loss being supportive of but not diagnostic for the diagnosis of NEC (Basturk *et al.* 2015, Konukiewitz *et al.* 2017, Bellizzi 2020*b*). Molecular pathology is also used to identify clinically actionable events (e.g. mismatch repair (MMR) deficiency/microsatellite instability, pan-TRK overexpression/*NTRK* fusion).

#### Biopsy of primary vs metastatic site

The purpose of the biopsy in high-grade NENs is to secure the diagnosis, assess the proliferation index (especially in G3 NET), assign site of origin if possible (in metastases of unknown primary), and identify clinically actionable molecular genetic events. Biopsy of either primary or metastatic tumor should achieve these goals and there was consensus that a biopsy of both the primary and a metastasis is not necessary. As systemic and biologic therapies are directed at metastatic disease, this is theoretically the optimal tissue to target, though most oncogenic drivers are stable in matched primary-metastatic pairs (Brannon *et al.* 2014). In general, there is no reason to re-biopsy NEC on disease progression as this does not generally change management (majority). It is reasonable to re-biopsy G3 NET patients, particularly if there is an escalation in the pace of the disease or if one lesion seems to be growing faster than others and the majority of the group agreed with this approach as it may impact treatment selection. It is very unusual for G3 NET to transform to NEC although there is consensus that this would certainly change how a patient would be managed.

#### Summary of recommendations

High-grade NENs include both G3 NET and NEC and Ki-67 IHC is indispensable to NET grading and is prognostic and predictive in NEC. Mutant-pattern p53 staining and/or Rb loss generally support a diagnosis of NEC over G3 NET. Biopsy of either primary or metastatic tumor is sufficient for diagnosis and biomarker testing.

### Imaging

#### Anatomical imaging

Ultrasound, computed tomography (CT), and magnetic resonance imaging (MRI) all have well-established roles for imaging NENs with CT, particularly multiphase CT, being the cornerstone of neuroendocrine imaging. This modality is well suited for the identification of the primary tumor, staging, and for response assessments (van Essen *et al.* 2014), but multiple studies have also shown that key characteristics can be used to differentiate high-grade lesions from their low or intermediate counterparts. Among characteristics associated with G3 lesions are larger size, ill-defined features, portal and arterial enhancement and textural analysis to name a few (Feng *et al.* 2014, Utsumi *et al.* 2015, Zamboni *et al.* 2017, Canellas *et al.* 2018, Guo *et al.* 2019). For pancreatic primaries, larger tumor size and main pancreatic duct obstruction are associated with G2/G3 tumors (Fujimori *et al.* 2016). Multiphase MRI is an acceptable alternative with advantages including the lack of ionizing radiation and better soft-tissue contrast than CT. As with CT, a variety of MRI features have been shown to differentiate grade, including the apparent diffusion coefficient and diffusion-weighted imaging (Lotfalizadeh *et al.* 2017, Kulai *et al.* 2018, Mebis *et al.* 2020).

#### Functional imaging

Functional (nuclear) imaging is essential for NENs as an adjunct to anatomical imaging. The options include ^111^In-pentetreotide, ^68^Ga-DOTATATE, ^68^Ga-DOTATOC, ^64^Cu-DOTATATE, and ^18^F-fluorodeoxyglucose (FDG). The first four are somatostatin analogs (SSAs) showing the somatostatin-receptor (SSTR) expression levels in tumors, while ^18^F-FDG is a glucose analog showing the glucose transporter levels in tumors (Hicks 2010, Hofman & Hicks 2012, Toumpanakis *et al.* 2014). Throughout the remainder of this article, these will generically be referred to as SSTR imaging and FDG imaging. Importantly, DOTATATE/DOTATOC positron emission tomography (PET) is superior to pentetreotide gamma imaging in several significant respects including resolution, radiation dose and imaging time. As such, wherever available, DOTATATE PET is the current agent of choice for SSTR imaging.

The added value of SSTR imaging over anatomical imaging includes identification of the primary when not already known, identification of additional lesions without clear anatomical correlates, and evaluation of SSTR expression which is important for therapeutic decision-making, especially for SSTR-directed therapy. SSTR expression is inversely correlated with tumor grade with SSTR expression being seen mainly in G3 NETs and sometimes with NEC (Kaemmerer *et al.* 2011, Majala *et al.* 2019). In 40.9% of patients, the treatment plan is changed after the scans, owing mainly to new, unexpected findings (Skoura *et al.* 2016). There was consensus among panelists that SSTR imaging should be obtained at baseline for all patients with a G3 NET as this will be required if ^177^Lu-DOTATATE is being considered as a treatment option (Sorbye *et al.* 2020). Conversely, the role of SSTR imaging for NEC is not clearly supported and there was consensus that baseline SSTR imaging should not be obtained for patients with a newly diagnosed NEC.

In contrast, the level of glucose transporters (FDG uptake) is directly correlated with tumor grade. Several studies have shown that the degree of FDG uptake can be used to differentiate G1/G2 from G3 tumors and that the degree of uptake is strongly correlated with prognosis (Panagiotidis & Bomanji 2014, Tomimaru *et al.* 2015, Majala *et al.* 2019). It should be the functional imaging of choice for NECs, and for NETs of all grades when SSTR expression is low or not present but there was lack of consensus that an ^18^F-FDG PET should be obtained at baseline for either patient with an NEC or a G3 NET.

The inverse correlation between SSTR imaging and FDG imaging across tumor grade suggests they provide complementary information with regard to sensitivity, tumor grade and aggressiveness (especially important for those with potentially heterogeneous disease), and for prognosis (Kayani *et al.* 2008, Partelli *et al.* 2014, Kubota & Okasaki 2014, Zhang* et al.* 2018). This is especially applicable to the G3 NETs given their inherent heterogeneity (Panagiotidis *et al.* 2017). There was a lack of consensus on whether discrepant findings between SSTR- and FDG-PET imaging would warrant additional biopsies as the more aggressive element of disease should be prioritized in determining treatment strategy.

#### CNS imaging

The incidence of brain metastases in extra-pulmonary high-grade NENs is less than 2% (Alese *et al.* 2019, Fottner *et al.* 2019). As such, routine brain imaging is not indicated for asymptomatic patients with NEC or in those with a low volume of systemic disease (consensus) (Alese *et al.* 2019, Fottner *et al.* 2019). Patients with a high burden of systemic disease may be at higher risk for developing brain metastases (Hlatky *et al.* 2004, Akimoto *et al.* 2016). For patients with a G3 NET, the majority of the panel felt that baseline CNS imaging was not indicated but there was a lack of consensus as to the role of baseline CNS imaging in all patients with a NEC.

#### Summary of recommendations

Anatomical imaging with CT/MRI (often multi-phase) is the mainstay of imaging across all grades of NEN and all patients with a G3 NET or NEC should have some form of anatomical imaging at the time of diagnosis. Functional imaging with SSTR- and/or FDG-PET are important adjuncts. SSTR-PET should be obtained for all patients with G3 NET, particularly for patients with metastatic disease, as there are associated treatment implications. FDG-PET is indicated if there is a lack of clarity between G3 NET and NEC and in cases where the tumor appears disproportionately aggressive. CNS imaging is indicated in symptomatic patients and may be considered in patients with high disease burden.

### Biomarkers and molecular characteristics

Biomarker levels, both hormonal and non-hormonal (chromogranin A) can be elevated in the presence of some G3 NENs although hormone-secreting G3 NENs are rare (Jensen *et al.* 2008, Metz & Jensen 2008, Reidy-Lagunes 2012, Castellano *et al.* 2015) No studies have demonstrated a clear correlation between levels of any biomarker and clinical and/or radiographic response to therapy. There was a lack of consensus that evaluation of biomarkers should be conducted during the initial assessment of patients with a G3 NET aside from patients with a clinical syndrome. For patients with a clinical hormonal syndrome, there was consensus that hormone levels tailored to the patient’s symptoms should be checked at diagnosis and throughout their treatment course. There was also consensusthat monitoring of biomarkers during treatment of patients with non-functional tumors would not change management, should not be used in isolation for treatment decision-making and that there is no role for biomarker evaluation at the time of diagnosis for NEC.

G3 NET and NECs are characterized by distinct molecular profiles, reflective of differing mechanisms of pathogenesis; however, our understanding of the treatment implications of these distinct mutational spectrums remains limited. Multiple sequencing efforts (whole-exome, whole-genome, next-generation) have identified an increased number of somatic mutations in the cell cycle regulator *CDKN1B*, chromatin remodeling genes (*MEN1, DAXX, ATRX*,* ARID1A*), DNA repair genes, and mammalian target of rapamycin (mTOR) pathway genes in well-differentiated NETs (Jiao *et al.* 2011, Scarpa *et al.* 2017, Raj *et al.* 2018). In regard to *DAXX* and *ATRX* specifically, although there are some conflicting data (Jiao *et al.* 2011, Raj *et al.* 2018), one of these genes appears to be more commonly mutated in G2 or G3 NET (70%) as compared to G1 NET (30%) (Singhi *et al.* 2017), and presence of a *DAXX* or *ATRX* mutation is associated with a significantly decreased disease-free survival (HR 4.69, *P* < 0.001). Another study similarly showed a poorer prognosis with an *ATRX* mutation (HR 16.982, *P* = 0.012) (Chou *et al.* 2018). G3 NET specimens are represented in very low numbers and are included in only a few of these studies, making this an area in need of further exploration.

Sequencing efforts in NEC have identified somatic alterations most commonly in *TP53* and in retinoblastoma (Rb) pathway genes, (McNamara *et al.* 2020). Up to 50% of extra-pancreatic NECs have mutations in *TP53* but *RB1* mutations are somewhat less common (Bergsland *et al.* 2016, McNamara *et al.* 2020). Mutations in *KRAS, TP53, BRAF* (especially V600E), and *APC* are commonly seen in colorectal NECs (Puccini *et al.* 2020, Chen *et al.* 2021, Lee & Sung 2021). Some studies of NEC have also identified tumor microsatellite instability (Yachida *et al.* 2012, Tang* et al.* 2016*b*, Girardi *et al.* 2017).

Molecular characterization in NEC of gynecologic origin suggests yet still different features, particularly in cervical primaries. Positivity for human papillomavirus (HPV) is known to be present in 85.6% of these cancers. Akin to NEC of the gastroenteropancreatic system, *Rb1* mutations are seen but in lower percentages and differ between HPV-positive and HPV-negative patients (4 and 32%, respectively). Mutations in *PIK3CA* (19.6%), *MYC* (15.5%), *TP53* (15.5%), and *PTEN* (14.4%) are also seen, as are tumors with intermediate or high tumor mutational burden (18.6%), at times also showing DNA MMR deficiency (Eskander *et al.* 2020).

Despite an improved understanding of NET and NEC genetics, current data are not strong enough to use genomic profiles for standard of care treatment selection. Studies evaluating the use of the targeted therapy everolimus in the setting of mTOR pathway alterations have not identified a neuroendocrine subgroup more likely to respond (Zatelli *et al.* 2016). Further study is clearly needed but the availability of therapeutics that are disease agnostic make next-generation sequencing (NGS) and an evaluation for DNA MMR deficiency reasonable; approximately 10% of NEC demonstrate MMR deficiency (Girardi *et al.* 2017), 0.31% of NENs have NTRK fusions (Solomon *et al.* 2020), and it has been reported that at least 20% of NECs have some form of druggable molecular alternation (Garcia-Carbonero *et al.* 2023). Rare cases of RET fusions have also been reported (Subbiah *et al.* 2022).

Of note, the earlier NEN sequencing efforts have been conducted in tumor tissue specimens and work on circulating tumor specimens is limited and investigational. In the CIRCAN-NEC pilot study, circulating tumor DNA (ctDNA) was evaluated during the course of a patient’s therapy and in the context of their mutational status (*BRAF, KRAS, RB*, etc). This study included 24 patients but demonstrated early findings that ctDNA may potentially be helpful in predicting prognosis and could aid in selecting the best therapy (Gerard *et al.* 2021). Circulating tumor cells (CTCs) have been identified in NENs and CTC presence has been associated with increased tumor burden and higher tumor grade (Khan *et al.* 2013, Zatelli *et al.* 2017, Rizzo & Meyer 2018). While still investigational, given the CTC presence in some NENs, at some point, molecular testing of CTCs may provide an opportunity to non-invasively identify clinically actionable events in G3 NETs and NECs.

#### Summary of recommendations

Initial and subsequent biomarker assessments should only be conducted for patients with a hormone-mediated clinical syndrome and should not be used in isolation for diagnosis or monitoring of either G3 NET or NEC. There was consensus from the group that NGS and MMR testing should be routinely conducted for patients with an NEC as this may identify targets for disease-agnostic treatments, but there was lack of consensus in regard to its use for G3 NETs. Blood collection for ctDNA and CTCs remains investigational and should be encouraged in the context of clinical trials.

### Studies to be done as part of initial assessment

Studies to be performed as part of the initial NEN evaluation have been provided by three other societies that have generated neuroendocrine guidelines. These include the National Comprehensive Cancer Network (NCCN) (Shah *et al.* 2021), the European Society of Medical Oncology (ESMO) (Pavel *et al.* 2020), and the European Neuroendocrine Tumor Society (ENETS) (Garcia-Carbonero *et al.* 2016, Sorbye *et al.* 2023). Based on these societal recommendations as well as the data presented earlier, we recommend the following be performed as part of the initial grade 3 NEN assessment (Table 1).

**Table 1:** Recommended assessments for a newly diagnosed grade 3 neuroendocrine neoplasm.

	G3 NET	NEC
Imaging	Multiphase, high-resolution CT or MRI with contrast	Multiphase, high-resolution CT or MRI with contrast
	SSTR-PET imaging	MRI brain for high systemic burden and/or if symptomatic
	FDG-PET if indicated	FDG-PET if indicated
		SSTR-PET imaging if indicated
Laboratory (if metastatic disease present)	MSI/MMR testing may be considered	MSI/MMR testing (should not delay initiation of treatment)
	Next-generation sequencing may be considered	Next-generation sequencing (should not delay initiation of treatment)
Endoscopy	Endoscopic evaluation (EGD, colonoscopy) for known or suspected GI primaries	Endoscopic evaluation (EGD, colonoscopy) if possible for known or suspected GI primaries – identification of primary should not delay treatment

## Management of localized disease

### Gastrointestinal G3 neuroendocrine tumors (NET)

#### Surgical resection

Outcomes for patients undergoing surgery for the locoregional disease are confounded by a limited number of studies that combine G3 NET and NEC, combine disparate primary tumor sites, and include patients with locoregional and advanced disease (Haugvik *et al.* 2016, Tang* et al.* 2016*b*, Dasari *et al.* 2018, Yoshida *et al.* 2019, Pommergaard *et al.* 2021). Nevertheless, median survival exceeding 43–55 months has been reported for G3 NET patients, suggesting favorable biology (Crippa *et al.* 2016, Tang* et al.* 2016*b*, Yoshida *et al.* 2019) and there was consensus from the group that surgery may be appropriate for locoregional control and possibly cure. The goals of surgery for patients with localized G3 NET are an oncologic resection of the primary tumor and regional lymph nodes. Since most patients with G3 NENs have lymph node metastases, a thorough lymphadenectomy is necessary. For pancreatic primaries involving the pancreatic body and tail, a distal pancreatectomy with en-bloc splenectomy is the recommended surgical approach (Nakakura 2018).

#### Radiation therapy, chemotherapy, and chemoradiotherapy

There are limited data regarding definitive, neoadjuvant, or adjuvant radiation therapy in the management of G3 NEN. Optimal concurrent chemotherapy is unknown; commonly used regimens include fluoropyrimidine-based treatment or platinum and etoposide. This treatment may be delivered as a neoadjuvant treatment for patients planned to undergo surgery, which is favored over delivery in the adjuvant setting based on extrapolation from data describing improved toxicity and efficacy with gastrointestinal adenocarcinoma treatment (Spitz *et al.* 1997, Sauer *et al.* 2012, Wong *et al.* 2015). The majority of the group felt that there is no role for neoadjuvant radiation or chemoradiation for patients with G3 NETs but there was a lack of consensus as to its use in the adjuvant setting, regardless of margin status following surgery. For patients with pancreatic NETs with adverse postoperative features, there may be a local control benefit (Contessa *et al.* 2009, Arvold *et al.* 2012, Zagar *et al.* 2012). This is an area where further investigation is sorely needed.

There are no data regarding the use of adjuvant chemotherapy for patients with a resected G3 NET despite their high risk of recurrence. The SWOG 2104 trial is addressing the role of postoperative adjuvant temozolomide and capecitabine in patients with a resected well-differentiated G3 NET of the pancreas, specifically those with a Ki-67 of <55% (NCT05040360).

### Gastrointestinal neuroendocrine carcinomas (NEC)

#### Role of surgery

Patients with NEC have an extremely poor prognosis with median survival in most cases of 13 months or less, even for patients with locoregional disease undergoing resection with curative intent (Smith & Reidy-Lagunes 2013, Basturk *et al.* 2014, Smith *et al.* 2014, Crippa *et al.* 2016, Fields *et al.* 2019, Yoshida *et al.* 2019, Ueberroth *et al.* 2021). Therefore, the role of surgery for G3 NEC is highly questionable. Although a preoperative diagnosis of a localized G3 NEC is rarely made, if indeed the diagnosis is known, the panel strongly recommends multidisciplinary evaluation and consideration of systemic therapy and possible definitive chemoradiation, particularly for surgeries with higher morbidity (esophageal, gastric, pancreatic, and rectal primaries) (Crippa *et al.* 2016, Deng *et al.* 2016). Both the ENETS and ESMO guidelines recommend surgery for patients with a localized NEC although both also indicate a role for chemotherapy and possibly radiation for some of these patients, particularly those with esophageal, anal, and some rectal primaries (Garcia-Carbonero *et al.* 2016, Pavel *et al.* 2020, Sorbye *et al.* 2023). An evaluation of the role of surgery in localized NEC in the United States carried out on data from the National Cancer Database (NCDB) suggests that for patients with stage I–II disease, surgery does confer a survival benefit and should be considered. This study, however, was conducted on data that did not adhere to current WHO criteria and Ki-67 data was not available to determine if these patients had G3 NET or NEC (Dasari *et al.* 2022). Another retrospective study of patients with localized poorly differentiated NEC showed a survival advantage for patients undergoing surgery but again, Ki-67 data were not available and it is unclear if patients were appropriately classified (Thornblade *et al.* 2021). Additional limited studies suggest definitive chemoradiation may yield favorable outcomes compared with surgery for esophageal and rectal primaries (Meng *et al.* 2013, Bertani *et al.* 2018, Dasari *et al.* 2022).

#### Radiation therapy, chemotherapy, and chemoradiotherapy

Distant progression is the most common site of failure for NEC, demonstrating the importance of systemic therapy. However, (chemo)radiation may provide durable local control for primary and metastatic disease (Chakravarthy & Abrams 1995). NECs have most commonly been treated with concurrent doublet platinum-based therapy (Meng *et al.* 2013) with four cycles (based on small-cell lung cancer data) (Faivre-Finn *et al.* 2017) or five to six cycles (based on gynecologic data) (Viswanathan *et al.* 2004) being the norm. When given alongside radiation, platinum and etoposide are the recommended radiosensitizing regimen (Yamashita *et al.* 2009, Meng *et al.* 2013, Katada *et al.* 2020). If the ability to deliver etoposide is hampered, carboplatin and paclitaxel is an acceptable alternative, based on small-cell lung cancer data (Groen *et al.* 1999). Several retrospective studies evaluating the benefits of adjuvant chemotherapy have yielded discordant results (Pellat *et al.* 2020, Mao *et al.* 2021, Schmitz *et al.* 2021). In the aforementioned NCDB study (Dasari *et al.* 2022) where surgery was recommended for very early disease, chemoradiation was found to likely be preferred over surgery for patients with primary tumors where a morbid surgery would be undertaken. Currently ongoing is the French NEONEC trial (NCT04268121) which is evaluating both neoadjuvant treatment with platinum and etoposide followed by surgery or chemoradiation as well as the role of adjuvant platinum and etoposide chemotherapy in patients with localized GI NEC. This prospectively collected data will be critical in guiding physicians as to how best to manage this unique patient population. It is the view of ENETS that adjuvant therapy following surgical resection of a localized NEC may be considered (Sorbye *et al.* 2023).

#### Summary of recommendations

There was an overall lack of consensus from the panel in regard to the role of surgery for NEC although it was more favorably viewed to pursue surgery following a course of systemic therapy and/or chemoradiation, particularly in exceptional responders or patients with very early disease. Following surgery or as definitive therapy, there was consensus that four to six cycles of platinum and etoposide with the inclusion of radiation at some point during the course of chemotherapy be considered and that capecitabine is an acceptable radiation sensitizer if consolidation chemoradiation is given following completion of four to six cycles of platinum and etoposide chemotherapy. There was a lack of consensusas to if chemoradiation should be used in the adjuvant setting for patients with a negative surgical margin although there was consensus that adjuvant chemoradiation is indicated following resection of a NEC with positive margins. There was consensus that patients with a resected NEC in a location not amenable to subsequent radiation should receive adjuvant chemotherapy.

### Surveillance assessments for localized disease

Surveillance studies to be performed for patients who have been treated for a localized NEN have also been provided in the guidelines of the aforementioned societies (NCCN, ESMO, and ENETS) (Garcia-Carbonero *et al.* 2016, Pavel *et al.* 2020, Shah *et al.* 2021). Based on these societal recommendations and in line with the data presented earlier, the majority of the panel agreed that the following surveillance studies be performed for patients who have been treated for a localized grade 3 NEN (Table 2).

**Table 2:** Surveillance assessments for patients treated for a localized grade 3 neuroendocrine neoplasm.

	G3 NET	NEC
Imaging	Multiphase, high-resolution CT or MRI with contrast every 3 months for 2–3 years, then every 6–12 months for at least 10 years	Multiphase, high-resolution CT or MRI with contrast every 3 months for 3 years, then every 6 months for years 4–5
	SSTR-PET imaging as indicated	MRI brain as indicated based on symptoms
	FDG-PET imaging as indicated	FDG-PET as indicated
		SSTR-PET imaging as indicated
Biopsy	Biopsy as indicated to confirm recurrence and/or to re-establish grade	Biopsy as indicated to confirm recurrence

### Localized gynecologic G3 neuroendocrine carcinoma

When it comes to the gynecologic tract, the vast majority will be NEC as opposed to NETs. If a NET is found in the gynecologic tract, metastatic disease from a gastrointestinal primary should be ruled out. Small-cell carcinoma is more frequently seen than large-cell NEC. NECs of the cervix predominate, found in descending order of frequency by those arising in the uterus, vulvo/vagina, and ovary. Due to the extreme rarity of these cancers (<300/year in the United States), multi-modal treatment recommendations have evolved by combining strategies for small-cell carcinoma of the lung and squamous cell/adenocarcinoma of the cervix. Prospective clinical trials, however, have not been performed in patients with these cancers.

For patients with disease confined to the cervix and <4 cm in size (FIGO stage IA1–IB2), surgery remains the first line of treatment. Standard surgery includes a radical hysterectomy and either sentinel lymph node biopsies or complete pelvic lymphadenectomy. In one large study, patients with early stage disease who did not undergo surgery had a hazard ratio for death of 4.74 compared to those who underwent surgery (Ishikawa *et al.* 2018). In a SEER database review, patients with early stage disease who underwent surgery had an odds ratio for recurrence of 0.62 compared to those who do not undergo surgery (Cohen *et al.* 2010). Although patients with NEC of the cervix were not included in the LACC study, which showed that minimally invasive radical hysterectomy had significantly higher recurrences and cancer-related deaths than open surgery, we recommend an open surgery as opposed to a minimally invasive approach (Ramirez *et al.* 2018). Fertility-sparing surgeries such as cone biopsy or radical trachlectomy should not be performed (Plante *et al.* 2011).

Postoperative chemotherapy is an integral part of adjuvant therapy for patients with early stage NEC of the cervix. Patients with stage IA2–IB2 disease who recur will have a distant component (i.e. outside the pelvis) 86% of the time (Zivanovic *et al.* 2009). The most common site of first recurrence is the lung followed by the liver and then the peritoneum, which further argues for systemic therapy (Stecklein *et al.* 2016). The addition of chemotherapy to surgery significantly reduces the risk of extrapelvic recurrence (OR for recurrence 0.37) (Ishikawa *et al.* 2018). Cisplatin and etoposide are the most commonly used therapeutic agents for adjuvant chemotherapy in patients with NEC of the cervix. In one study, patients who received chemotherapy other than cisplatin and etoposide had a hazard ratio for recurrence of 3.42 (Pei *et al.* 2017). Those patients who did not get any adjuvant chemotherapy had a hazard ratio for recurrence of 5.4. Furthermore, patients who received ≥5 cycles of cisplatin and etoposide had an improved 5-year recurrence-free survival. By consensus, we, therefore, recommend six cycles of adjuvant cisplatin and etoposide for patients with early stage disease who undergo surgery as primary therapy.

The benefit of radiation in addition to chemotherapy and surgery is less clear. Pelvic radiation after surgery does reduce pelvic recurrence. Patients receiving postoperative pelvic radiation have a 13–16% chance of pelvic recurrence vs a 25–31% chance if no radiation is received (Chen *et al.* 2015, Ishikawa *et al.* 2018). However, although postoperative pelvic radiation reduces pelvic recurrences, no studies have shown that it improves survival. This may be because studies are inadequately powered to show such a difference due to small sample sizes or due to the high risk of distant recurrence (liver, lung), local control of the pelvis does not improve survival. As there are no definitive data showing a survival benefit for postoperative pelvic radiation, we recommend postoperative radiation in addition to chemotherapy as adjuvant therapy in patients with high-risk factors such as tumors >2 cm, deep stromal invasion and/or lymphovascular space invasion (consensus).

Guidelines from the Society of Gynecologic Oncology and the Gynecologic Cancer Intergroup allow for neoadjuvant chemotherapy and surgery for cervix confined lesions >4 cm in size (FIGO stage IB3) or tumors with extension onto the upper vagina (stage IIA2) (Gardner *et al.* 2011, Satoh *et al.* 2014); however, there does not appear to be sound rationale for this approach. First, this strategy is not utilized for small-cell carcinoma of the lung or for squamous or adenocarcinoma of the cervix. Second, the likelihood of metastatic disease with tumors >4 cm is high. Finally, radiation therapy provides good pelvic control with pelvic recurrences only 10–20% with chemoradiation therapy (Hoskins *et al.* 2003, Stecklein *et al.* 2016). Recurrence rates for patients undergoing this approach are quite high at 79% (Bermudez *et al.* 2001, Lee *et al.* 2008, Nasu *et al.* 2011, Dongol *et al.* 2014). For those reasons, by consensus, we recommend chemoradiation followed by chemotherapy and not surgery for patients with stage IB3–IIA2 disease.

#### Summary of recommendations

For patients with early stage NEC of the cervix (FIGO stage IA1–IB2), we recommend radical hysterectomy and sentinel lymph node biopsy or complete pelvic lymphadenectomy with postoperative adjuvant therapy inclusive of pelvic chemoradiation with cisplatin and etoposide (rwo cycles) followed by an additional four cycles of cisplatin and etoposide for a total of six cycles. Patients with stage IB3–IIA2 disease should be treated with platinum/etoposide chemoradiation (two to three cycles) followed by an additional three to four cycles of chemotherapy (to complete a total of six cycles of chemotherapy) but not surgery given the high risk of distant recurrence.

## Treatment of metastatic G3 NET

To date, there is a paucity of prospective trials evaluating systemic therapy for G3 NETs and the majority of available studies are entirely retrospective in nature, small, and of low quality. Considerable controversies exist regarding the choice of systemic therapy in first-line therapy and beyond (Sonbol & Haldanarson 2019, Sorbye *et al.* 2019). There is substantial heterogeneity among the published studies in terms of the populations studied, the regimens used, and the methodology used for response assessment. Given the relatively recent recognition of G3 NETs as a subset of G3 NENs, many older studies do not adequately distinguish G3 NETs from NECs (for development of these recommendations, studies that did not analyze outcomes of the subset of patients with G3 NETs were excluded). Interpretation of these results is further limited due to G3 NETs constituting only a small proportion of the total study populations often without confirmatory expert pathological review prior to inclusion to firmly establish grade and differentiation. Common response criteria such as RECIST were uncommonly applied.

In the absence of higher-quality data, there was consensusamong the panel members that it would be appropriate to apply knowledge from G1 and G2 NETs to G3 NETs, particularly for those tumors exhibiting favorable tumor biology. The majority of the group felt that surgical debulking of patients with G3 NETs may also be appropriate.

### Liver-directed therapy

The presence of liver metastases has been shown to negatively affect the quality of life and prognosis of patients with NETs (Janson *et al.* 1997, Yao *et al.* 2007, Frilling *et al.* 2010). Liver-directed therapies using thermal ablation and/or intra-arterial embolization are commonly used for non-surgical NET patients with the liver-dominant disease to improve survival and quality of life, especially in patients with hormonal syndromes (Touzios *et al.* 2005, Kennedy 2016, Al-Toubah *et al.* 2019). Current guidelines endorse hepatic arterial embolization for symptomatic or progressive hepatic metastases, without recommendations among the different embolotherapies (chemoembolization, radioembolization, or bland embolization) (Pavel *et al.* 2016, 2020, Strosberg *et al.* 2017). Despite the lack of completed randomized trials comparing these embolization modalities, they have shown good clinical, biochemical, and morphological responses when liver tumor burden is beyond ablative therapies (Zappa *et al.* 2012). The ongoing RETNET trial (Randomized Embolization Trial for Neuroendocrine Tumor Metastases to the Liver, NCT02724540) is comparing bland embolization to conventional chemoembolization in patients with well-differentiated tumors, including G3 NET, and will provide much needed prospective data in this space.

There is a paucity of data in regard to the role of liver-directed therapy in G3 NET (Del Prete *et al.* 2014, Coriat *et al.* 2016, Chen *et al.* 2017). In one larger multi-institutional series, the number of G3 NET patients reached only 20 (13% of evaluated patients) – chemoembolization (*n* = 12), radioembolization (*n* = 7), and bland embolization (*n* = 1) (Chen *et al.* 2017). Patients with G3 NET were found to have a worse prognosis with shorter survival after embolization than patients with G1/G2 tumors (Chen *et al.* 2017).

From an expert opinion point of view, liver-directed therapy for metastatic G3 NET is considered a valid therapy similar to G2 NET if Ki-67 is <55%. Clinical considerations should be taken into account including rate of progression, tumor proliferative activity, and the overall disease course of the patient. The technique used is left to the discretion of the treating physician. In G3 NET with Ki-67 >55%, and in patients with extrahepatic metastases, especially bone, a systemic treatment is preferred. There was consensus that the use of liver-directed therapy in G3 NET is warranted; however, the ideal type of embolotherapy remains unclear.

#### Somatostatin analog therapy

SSAs are routinely used for the control of hormone-mediated symptoms in low-grade NETs (Rubin *et al.* 1999, Fisher *et al.* 2018). Functional G3 NETs are relatively uncommon, but up to a quarter of patients demonstrate hormone-mediated symptoms (Sorbye *et al.* 2018). Very little data exist to guide our approach to the use of SSAs in SSTR-positive G3 NETs from either symptom control or cancer growth standpoint, but data suggest that up to 88% of tumors express SSTR by imaging (Sorbye *et al.* 2018). A few small series lend support for activity in G3 disease. In a study of 14 G3 NET patients (median Ki-67 25%) treated with SSA monotherapy, half of the patients experienced stable disease or better (McGarrah* et al.* 2020*a*). The median PFS was 4.4 months. Three retrospective studies assessing the efficacy of SSA therapy in G3 NETs showed a median PFS of 4–8 months (Faggiano *et al.* 2016, de Mestier *et al.* 2021, Lithgow *et al.* 2021). None of the current NET guidelines addresses the use of SSAs for G3 NETs (Boudreaux *et al.* 2010, Pavel & de Herder 2017, Pavel *et al.* 2017).

#### Summary of recommendations

Extrapolating from well-differentiated G1/G2 NET, there was consensusfrom the panel that a trial of SSA therapy as the initial front-line therapy for tumor control in patients with a G3 NET with favorable biology is reasonable. The majority of the panel felt that this approach is only appropriate if the patient has positive SSTR imaging. Restricting the use of SSAs to SSTR-positive G3 NET with a relatively low Ki-67 makes the most sense, recognizing that the anticipated benefit is likely to be less durable than in G1/G2 NETs.

#### Front-line chemotherapy

All the studies reporting on first-line therapies for G3 NET were hampered by one or more of the limitations discussed earlier. The majority of front-line studies have evaluated cytotoxic chemotherapy, patients with a median Ki-67 ranging from 21 to 47% and the majority have assessed pancreatic NENs (Brixi-Benmansour *et al.* 2011, Ferrarotto *et al.* 2013, Hijioka *et al.* 2017, Jia *et al.* 2017, Girardi *et al.* 2017, Roquin *et al.* 2018, Apostolidis *et al.* 2018, Rogowski *et al.* 2019, Apostolidis *et al.* 2021, de Mestier *et al.* 2021, Lithgow *et al.* 2021, Liu *et al.* 2021). Platinum (cisplatin/carboplatin)-based regimens had response rates ranging from 0 to 38% with PFS ranging from 2.6 to 8.9 months (Velayoudom-Cephise *et al.* 2013, Heetfeld *et al.* 2015, Girardi *et al.* 2017, Hijioka *et al.* 2017, Jia *et al.* 2017, Li *et al.* 2017, Roquin *et al.* 2018, Apostolidis *et al.* 2021, Liu *et al.* 2021). The NORDIC NEC study retrospectively evaluated outcomes in 196 patients with gastroenteropancreatic NENs treated with platinum-based therapies of which 24 were G3 NETs. These patients had an overall response rate of 24% with a PFS of 5 months (as compared to an overall response rate of 44% for NEC with a Ki-67 of >55% and PFS of 5 months in NECs) (Sorbye *et al.* 2013). Together, these data suggest that platinum-based regimens have modest activity in G3 NETs. However, these data need to be interpreted with caution given the lack of information as to whether these regimens were selected due to concern about more aggressive NENs in these patients consistent with NECs. Temozolomide, especially in combination with capecitabine (CAPTEM), was the next most commonly reported regimen after platinum-based regimens with response rates ranging from 12 to 38% and PFS ranging from 6.7 to 15 months (Sorbye *et al.* 2013, Girardi, Silva *et al.* 2017, Jia *et al.* 2017, Sahu *et al.* 2018, Rogowski *et al.* 2019, Sahu *et al.* 2019, Chan *et al.* 2021, de Mestier *et al.* 2021, Lithgow *et al.* 2021). CAPTEM as an alternative front-line option was prospectively evaluated in the EA2142 clinical trial in which 63 patients with a non-small-cell gastroenteropancreatic G3 NEN (21 G3 NET, 36 NEC, 5 unknown) received either platinum and etoposide or CAPTEM. PFS with CAPTEM was not superior to platinum and etoposide at 3.45 vs 5.36 months. OS with CAPTEM was slightly better than platinum and etoposide at 12.6 vs 10.6 months (Eads *et al.* 2022). Studies evaluating FOLFOX reported a response rate of 64% in one study (Apostolidis *et al.* 2021) and two studies reported PFS of 8.6 and 10.8 months (Apostolidis *et al.* 2021). A single-center study reported similar findings but substantially shorter PFS with platinum/etoposide regimens than FOLFOX, 2.9 vs 13 months, respectively (Liu *et al.* 2021).

#### Summary of recommendations

G3 NET with Ki-67 <55–60% may have modest responses to platinum-based first-line therapies and the majority of the panel felt that this was not an appropriate front-line chemotherapy regimen in this setting. The majority of the group felt that CAPTEM was an appropriate front-line option particularly for patients with pancreatic primaries although patients with a G3 NET of the bowel may also benefit (despite data for this regimen in bowel primaries being less strong). The majority of the group felt that for pancreatic and bowel primaries, a fluoropyrimidine plus oxaliplatin regimen (either FOLFOX or CapeOx) is also appropriate.

#### Chemotherapies for second-line and beyond

Several small studies have evaluated the role of temozolomide-based therapy in the second line, most commonly as CAPTEM (Rogowski *et al.* 2019, Sahu *et al.* 2019, Chan *et al.* 2021). Most patients reported in these studies had a pancreatic primary and were previously treated, usually with a platinum-based regimen. Objective response rates ranged from 47 to 52% by RECIST and median PFS and median OS ranged from 6 to 15 months and 24 to 30 months, respectively. Multiple other chemotherapy regimens have been evaluated in small studies, including FOLFOX/CAPOX, FOLFIRI, and streptozocin-based and gemcitabine-based regimens, but the small sample sizes and the heterogeneity of the studies preclude recommendations for use (Cassier *et al.* 2009, Hentic *et al.* 2012, Ferrarotto *et al.* 2013, Dussol *et al.* 2015). One study found that among various cytotoxic regimens used in the second-line setting, FOLFOX yielded the longest PFS (13.9 months), while the PFS with CAPTEM and FOLFIRI was 7.7 and 2.4 months, respectively (Apostolidis *et al.* 2021). As such, CAPTEM is a reasonable choice for second-line therapy and beyond if not used in first line, with FOLFOX and FOLFIRI being alternative options.

#### Targeted therapies

Targeted therapy in both G3 NET and NEC is not well established with only a few retrospective studies describing the use of everolimus and sunitinib in patients with pancreatic G3 NETs (Panzuto *et al.* 2017, Mizuno *et al.* 2018, Pellat *et al.* 2018). Based on consensus, it is acceptable to consider everolimus for patients with progressive and refractory high-grade NETs of both pancreatic and bowel origin as well as sunitinib for G3 pancreatic NET, particularly in patients with favorable biology. Given the absence of good quality evidence and in light of the availability of agnostic targeted therapies (Drilon *et al.* 2018), the panel encourages the use of NGS for patients with G3 NETs as another tool to guide therapy in the refractory setting, especially if a clinical trial is not available.

#### Peptide receptor radionuclide therapy

Peptide receptor radionuclide therapy (PRRT) is approved in the United States for the treatment of somatostatin receptor-positive gastroenteropancreatic NETs, including foregut, midgut, and hindgut tumors. Although no randomized trial has established the role of PRRT in patients with G3 NENs, a few studies show encouraging results in patients with G3 NETs, reporting objective and durable responses ranging from 35 to 42% and a PFS ranging from 9 to 14 months (Thang *et al.* 2018, Zhang *et al.* 2019, Carlsen *et al.* 2019, Raj *et al.* 2022). The randomized clinical trial NETTER-2 evaluated the role of PRRT in G2/3 gastroenteropancreatic NETs with a Ki-67 index of ≥10 to ≤55% and completed accrual in late 2022 (NCT03972488). The COMPOSE trial is investigating ^177^Lu-Edotreotide vs best standard of care in aggressive G2/G3 gastroenteropancreatic NET (NCT04919226). Based on consensus, it is reasonable to consider PRRT in patients with progressive G3 NET showing homogeneously high (avidity greater than liver) SSTR expression by imaging.

#### Immunotherapy

Immunotherapy has not been shown to be active in NETs as suggested by the KEYNOTE-158 trial. In this trial, an overall response rate of 3.7% was observed with zero complete responses and four partial responses (Strosberg *et al.* 2020). A few single-arm phase II trials in G3 NENs suggest similar outcomes. A joint analysis of two phase II studies that enrolled 29 patients with previously treated metastatic G3 NENs to receive pembrolizumab showed limited activity with single-agent therapy with a response rate of only 3.5% (Vijayvergia *et al.* 2020). Avelumab was studied in G3 NENs through the AVENEC trial and included 10 patients with a G3 NET. The response rate was 6.9% in the entire cohort and PFS was similar for G3 NET as well as NEC (Fottner *et al.* 2019). The only trial to demonstrate the benefit of immunotherapy is a phase II trial of JS001 in 21 patients with NENs, 3 of which were G3 NET. Two of the G3 NET patients achieved a partial response and a response rate of 19% was observed amongst the whole cohort. Unfortunately, these responses did not translate into improvement in PFS (median PFS 2.8 months) (Zhang* et al.* 2018*a*).

Combination therapy with nivolumab and ipilimumab has shown promising activity in G3 NENs but the proportion of G3 NET vs NEC included is unknown (Patel *et al.* 2020). The recently reported DUNE study of durvalumab plus tremelimumab in different neuroendocrine cohorts included 33 patients with G3 NEN, including 15 patients with G3 NET. Overall, the G3 NEN cohort had a modest immune-related overall response rate of 9.4% and a 9-month OS of 36.1% (Capdevila *et al.* 2021). One-third of patients with G3 NEN had OS greater than 1 year, including patients with both G3 NET and NEC. The role of immunotherapy in G3 NET is not supported by data at this time and further prospective trials are needed.

#### Surgical debulking

Studies evaluating the outcomes of patients with metastatic G3 NEN who have undergone surgical resection/debulking have been limited by small numbers and the inclusion of patients with both G3 NET and NEC. There is evidence suggesting acceptable outcomes following liver debulking in select patients with G3 NET, particularly those with more favorable biology. A retrospective study of 32 patients with G3 NEN who underwent surgical and/or radiofrequency ablation of liver metastases with curative intent showed a median PFS of 8.4 months (95% CI: 20.6–51.3 months), OS of 35.9 months (95% CI: 20.6–51.3 months) and a 5-year OS of 43% (Galleberg *et al.* 2017). Factors associated with improved OS were Ki-67 <55% and receiving adjuvant chemotherapy. A retrospective analysis from Japan included 63 patients with G3 NEN and found that surgery and low Ki-67 value (<52%) were independent factors associated with improved survival (Asano *et al.* 2020). There was no difference in survival between those undergoing surgery vs chemotherapy in patients with a higher Ki-67 (≥52%). Morphologic differentiation as defined by WHO 2017 criteria showed no association with OS. Although selection bias may influence results, additional small retrospective studies have also indicated a potential survival benefit in patients with G3 NEN undergoing aggressive locoregional therapy (Du *et al.* 2015, Crippa *et al.* 2016). Overall, the majority of the panel felt that surgical debulking for patients with a metastatic G3 NET was appropriate, particularly for those with favorable biology, but that this should be determined within the context of a multidisciplinary discussion.

## Treatment of metastatic GI NEC

### Liver-directed therapy

Please see the liver-directed therapy section earlier for details regarding this therapy. No data exist that would justify the use of liver-directed therapy in NEC. However, there was a lack of consensus from the group in regard to its role in this patient population.

### Front-line chemotherapy

For the past 30 years, NEC has been treated akin to small-cell lung cancer. Early studies by Moertel and Mitry were the first to report on the favorable response rates of NECs with combined etoposide and cisplatin chemotherapy (Moertel *et al.* 1991, Mitry *et al.* 1999), although the response was not measured using conventional techniques. The largest cohort of advanced GI NEC patients reported is from the Nordic group (Sorbye *et al.* 2013). This retrospective study reported detailed data on treatment, outcomes, and survival. A total of 252 patients received palliative chemotherapy with platinum and etoposide chemotherapy. Responses were seen in 31% of patients and stable disease in 33% of patients. Patients with a Ki-67 < 55% had a lower response rate than patients with a Ki-67 ≥ 55% (15 vs 42%, *P* < 0.001) but better survival than patients with a Ki-67 ≥ 55% (14 vs 10 months, *P* < 0.001). It was also found that cisplatin could probably be replaced by the less toxic carboplatin, as the two compounds were comparable in efficacy. The addition of immunotherapy to platinum/etoposide has been shown to be superior to chemotherapy alone in small-cell lung cancer (Horn *et al.* 2018, Paz-Ares *et al.* 2019) and an ongoing SWOG trial is evaluating platinum/etoposide with or without atezolizumab in NEC (NCT05058651).

#### Summary of recommendations

These published reports, as well as the consensus opinion of the panel, recommend that patients with metastatic GI NEC be given palliative platinum and etoposide chemotherapy. The majority of the group felt that this regimen may be used for six cycles or longer provided there is no disease progression and the patient is tolerating treatment well. Immunotherapy should not be added to this regimen at this time and we await the results of the ongoing platinum/etoposide with or without atezolizmab trial (NCT05058651).

### Alternative front-line therapies

The combination of cisplatin and irinotecan (IP) has demonstrated at least equivalent efficacy as cisplatin and etoposide (EP) in advanced NEC of the intestinal system (Morizane *et al.* 2022). In the JCOG1213 randomized phase III trial, 170 patients with NEC received either EP or IP and no significant difference was seen in median OS (12.5 months with EP, 10.9 months with IP, HR 1.042, *P* = 0.797) or PFS (5.6 months with EP, 5.1 months with IP). Response rates were also similar (54.5% with EP, 52.5% with IP). IP was associated with less hematologic toxicity, making this a reasonable alternative front-line therapy. Similar results were observed in a smaller randomized phase II trial (Zhang *et al.* 2020) as well as several retrospective NEC trials (Okita *et al.* 2011, Nakano *et al.* 2012, Lu *et al.* 2013, Ramella Munhoz *et al.* 2013, Okuma *et al.* 2014, Yamaguchi *et al.* 2014). The triplet chemotherapy regimen of carboplatin, etoposide and paclitaxel did not show superiority to dual-agent therapy and was associated with higher rates of grade 3/4 toxicity (Hainsworth *et al.* 2006). Temozolomide and capecitabine have been evaluated as detailed in the G3 NET section (Eads *et al.* 2022).

In the front-line setting, capecitabine plus oxaliplatin (CAPOX) yielded a response rate of approximately 20% and 5-fluorouracil, leucovorin, oxaliplatin, and irinotecan (FOLFIRINOX) showed a near complete response in a patient with pancreatic NEC (Bajetta *et al.* 2007, Ferrarotto *et al.* 2013, Zhu *et al.* 2015). Although these results are encouraging, the studies are limited by small patient numbers and a lack of information regarding tumor differentiation. FOLFIRINOX is being compared prospectively to platinum/etoposide in the FOLFIRINEC trial, a randomized phase II trial (NCT04325425) (Hadoux *et al.* 2021).

#### Summary of recommendations

Cisplatin plus irinotecan is associated with response rates and PFS durations comparable to cisplatin plus etoposide, particularly for NEC arising in the GI tract or pancreas. There was consensus that platinum plus irinotecan is a reasonable alternative front-line chemotherapy option for NEC.

### Second- and third-line treatment options

Options for salvage therapy in patients with NEC have not been rigorously evaluated. There was consensus from the group that retreatment with platinum-based therapy can be considered in patients who experience a durable response to therapy and progression during a break from treatment. Several studies have also evaluated alternative regimens in patients whose disease has progressed on platinum-based therapy. A retrospective study of 64 patients with progressive NEC receiving second-line therapy of any sort after first-line platinum/etoposide showed limited efficacy of any second-line therapy and short survival (McGarrah* et al.* 2020*b*). The median PFS and OS were 2.3 and 6.2 months, respectively, and no particular regimen appeared superior to another. The recent phase II PRODIGE 41-BEVANEC trial randomized 133 patients to receive FOLFIRI with or without bevacizumab. The trial did not show any benefit for the addition of bevacizumab to chemotherapy but did report a 6-month OS rate of 53% (Walter *et al.* 2023). While this study did not compare FOLFIRI to an alternative cytotoxic backbone, the survival rate was favorable, making FOLFIRI an acceptable second-line treatment option with some groups suggesting this as a favored approach (Garcia-Carbonaro *et al.* 2023). In addition to irinotecan-based regimens, oxaliplatin-based therapy is also an option with retrospective data showing response rates of 20–30% with FOLFOX and CAPOX (Hentic *et al.* 2012, Hadoux *et al.* 2015, Heetfeld *et al.* 2015, Walter *et al.* 2017). Although CAPTEM has been evaluated in patients with G3 NENs, response and survival have been correlated with a Ki-67 index of <60%; there is limited data regarding the efficacy of CAPTEM specifically in patients with NEC (Welin *et al.* 2011, Owen *et al.* 2017, Chan *et al.* 2021). A second-line study of dacarbazine or temozolomide-based therapy reported a median PFS of 3 months and median OS of 7.2 months (Couronne *et al.* 2020). Other regimens such as topotecan and anthracyclines have been studied yielding similar disappointing results, sometimes with substantial toxicity, and are generally not recommended (Olsen *et al.* 2014, Apostolidis *et al.* 2016, Munker *et al.* 2020). The NET-02 trial was a randomized phase II study evaluating liposomal irinotecan (Nal-IRI) and 5-fluorouracil vs docetaxel with a primary endpoint of 6-month PFS rate. Although Nal-IRI/5-FU met the primary endpoint with a 32% 6-month PFS rate, both regimens had short median PFS of 3 and 2 months, respectively (Craig *et al.* 2020, McNamara *et al.* 2022). Clearly prospective trials in the second-line NEC space are needed. The SENECA trial is a randomized phase II trial evaluating CAPTEM vs FOLFIRI with a primary endpoint of disease control rate and safety (NCT03387592) (Bongiovanni *et al.* 2018).

#### Summary of recommendations

Depending on the timeline, re-treatment with platinum and etoposide is an option. FOLFIRI is a second-line option with the most evidence; however, additional options include oxaliplatin- or temozolomide-based regimens.

### Immunotherapy

Single-agent PD-1/PD-L1 inhibitor therapy has shown disappointing results in NEC as noted earlier. The only outlier was a phase II trial of JS001 in 21 patients with NENs, 15 of which were NECs (Zhang* et al.* 2018*b*). The response rate among NEC patients was 13% and unfortunately, no improvement in PFS was observed (median PFS 2.8 months).

In terms of combination therapy, a phase II basket study of ipilimumab (CTLA-4 inhibitor) and nivolumab (PD-1 inhibitor) in rare cancers (DART trial) suggested that dual checkpoint inhibition may yield a higher response rate than anti-PD-1 monotherapy in patients with G3 NENs. In a cohort of 18 patients with non-pancreatic NENs, a response rate of 44% and a median PFS of 4 months were observed. In a cohort of DART patients with high-grade NENs, including unknown, pancreas, GI, and cervical primaries, the response rate was 26%, including durable responses in some patients (Patel *et al.* 2021). Detailed information regarding the differentiation/grade of the bowel primaries was not reported, but all tumors were microsatellite stable (Patel *et al.* 2021). In yet another basket trial evaluating ipilimumab and nivolumab in rare cancers, combination immunotherapy was shown to have activity in subgroups of patients with advanced NENs. Objective responses were seen in 31% of the 13 patients with G3 disease (Klein *et al.* 2020). In the DUNE study, the combination of durvalumab (PD-L1 inhibitor) and tremelimumab (CTLA-4 inhibitor) in four NEN cohorts, including G3 NET/NEC, demonstrated a very modest response rate with the immune-related response rate of 9.4% in the G3 NEN cohort and a 9-month OS of 36.1% (Capdevila *et al.* 2021). PD-L1 expression was not found to enrich for response to immunotherapy. One of the largest trials specifically evaluating NEC was the phase II NIPINEC trial where 93 patients with gastroenteropancreatic NEC and 90 patients with large-cell NEC of the lung were randomized to nivolumab vs nivolumab plus ipilimumab in the second or third line setting. The overall response rate at 8 weeks and the median OS in the nivo vs combination therapy group were 7.5% and 7.2 months vs 14.9% and 5.8 months, respectively (Girard *et al.* 2021). Lastly, two recent and partially overlapping retrospective studies evaluated the role of immunotherapy in patients with refractory NEC (Al-Toubah *et al.* 2021, Gile *et al.* 2021). Immune checkpoint inhibitor monotherapy was ineffective but dual therapy yielded modest response rates of 13–15% and PFS was short at 1 to 3.5 months. Recent guidance from ENETS indicates that most patients with NEC do not benefit from immune checkpoint inhibitor therapy and that it should not be used in routine practice but may be considered in instances where patients have DNA MMR deficiency or high tumor mutational burden (Sorbye *et al.* 2023).

#### Summary of recommendations

Current data do not support the use of single-agent immune checkpoint inhibitors in G3 NECs. Preliminary data point to the role of dual immunotherapy and the majority of the group felt that this is a reasonable second line (and beyond) treatment option; however, data are sparse and clinical trial enrollment is encouraged. There was consensus that single- or dual-agent immunotherapy is appropriate in patients with DNA MMR deficiency or high tumor mutational burden, given the agnostic drug approvals of pembrolizumab (DNA MMR deficiency or high tumor mutational burden) and dostarlimab-gxly (DNA MMR deficiency).

### Somatostatin analogs

Traditionally reserved for well-differentiated NETs, SSAs are not thought to have a role in controlling tumor growth in NECs. In a study of 28 patients with G3 NENs receiving an SSA, G3 NETs were significantly more likely to be associated with positive SSTR imaging compared to NEC (88 vs 50% respectively) and demonstrated a better median OS (41 vs 17 months, respectively) (Velayoudom-Cephise *et al.* 2013). The ENETS guideline panel specifically notes that there are no data to support the use of SSAs in patients with SSTR-positive GI and pancreatic NECs, and neither the NANETS nor NCCN guidelines advocate for the use of SSAs in this setting (Strosberg *et al.* 2010, Garcia-Carbonero *et al.* 2016, Shah *et al.* 2021). As such, we do not recommend SSAs for tumor control in NEC.

### PRRT and targeted therapies

There is no data to support the use of PRRT in NEC. Given the expected low SSTR expression in this group, it is unlikely to have an effect. Similarly, data supporting the use of targeted therapies in NEC is lacking. Reports about BRAF inhibitors (+/− MEK inhibitors) in BRAF-mutated colorectal NECs are available (Klempner *et al.* 2016, Burkart *et al.* 2018); however, the use of BRAF inhibitors, MEK inhibitors, everolimus, and sunitinib are not approved for treatment of NEC. Targeted therapies with disease agnostic indications (immune checkpoint inhibitors for microsatellite unstable/deficient DNA MMR or high tumor mutational burden; larotrectinib or entrectinib for tumors with NTRK fusions; selpercatinib or pralsetinib for tumors with RET fusions) are a reasonable consideration for NEC patients harboring the appropriate molecular abnormalities but are rare.

### Role of surgery

Given the generally aggressive nature of NEC, surgical debulking is not performed. The importance of distinguishing G3 NET from NEC was highlighted in a retrospective multicenter analysis from Japan that evaluated the outcomes of 67 patients with G3 NEN of pancreatic origin (Yoshida *et al.* 2019). Patients with a pancreatic G3 NET who had undergone surgery had improved survival compared to those who had not; however, this was not the case for patients with pancreatic NEC. There was consensus from the group that there is no role for surgical debulking of patients with metastatic NEC.

### Monitoring assessments for metastatic disease

Given the relatively aggressive nature of high-grade NENs, regular assessment of disease status during treatment is important. Monitoring assessments have been recommended within the NCCN, ESMO, and ENETS NEN guidelines as previously mentioned (Garcia-Carbonero *et al.* 2016, Pavel *et al.* 2020, Shah *et al.* 2021). In line with these guidelines and based upon our review of the data, the majority of the panel recommends the following monitoring assessments be performed for patients receiving treatment for a metastatic grade 3 NEN (Table 3).

**Table 3:** Monitoring assessments for patients being treated for a metastatic G3 neuroendocrine neoplasm

	G3 NET	NEC
Imaging	Multiphase, high-resolution CT or MRI with contrast every 3 months (every 6 months if more indolent disease)	Multiphase, high-resolution CT or MRI with contrast every 2–3 months
	SSTR-PET imaging as indicated	MRI brain if high systemic disease burden or if symptomatic
	FDG-PET as indicated	FDG-PET as indicated
		SSTR-PET imaging as indicated
Biopsy	As indicated if pace of progression suggests a change in biology	Not indicated

## Treatment of metastatic gynecologic G3 NEC

Locally advanced disease, defined as a disease limited to the pelvis +/− aortocaval nodal disease (FIGO stages IB3–IVA) may be treated with intent to cure. Although some may be concerned with the use of radiation therapy concurrently with cisplatin and etoposide, studies have shown this is safe and tolerable and this approach has become the standard of care (Hoskins *et al.* 2003). Radiation can be started with cycle 1 of cisplatin and etoposide, utilized as ‘sandwich’ therapy (i.e. in the middle of chemotherapy), or added to the final two cycles of chemotherapy. As is the case with early stage disease, it is important to complete six cycles of chemotherapy if tolerated. Studies have shown improved disease-free survival and OS when ≥5 cycles are given (Wang *et al.* 2012). For metastatic disease to liver, lungs, or peritoneum (stage IVB), palliative chemotherapy with cisplatin and etoposide is recommended.

For patients with small-cell carcinoma of the cervix, commonly used second-line and beyond cytotoxic chemotherapy agents include topotecan, irinotecan and taxanes. A single institution retrospective analysis demonstrated superior PFS in patients with advanced cervical small-cell carcinoma receiving the combination of topotecan, paclitaxel, and bevacizumab (median PFS 8 vs 4 months for other regimens) (Frumovitz *et al.* 2017).

## Management of brain metastases

Given the low rate of intracranial metastases in patients with extra-pulmonary NENs, prophylactic cranial irradiation is not a recommended treatment strategy. General principles surrounding the management of brain metastases are recommended; patients with ≤4 metastases should be treated with stereotactic radiosurgery (SRS) while patients with more advanced CNS disease may be considered for SRS or whole brain radiation.

## Cardiac considerations

G3 NENs rarely result in functional syndromes, and therefore, there is little need for cardiac monitoring for carcinoid heart disease in this patient population (Sorbye *et al.* 2018). G3 NECs rarely if ever produce serotonin and among the G3 NETs, many are of pancreatic origin and therefore unlikely to produce serotonin (Phan *et al.* 2017, Tsoukalas *et al.* 2017, Zavras *et al.* 2017). While G3 NETs of small bowel primary are rare, they can be associated with carcinoid syndrome and therefore, carcinoid heart disease among all patients with G3 NENs is expected to be rare. Routine cardiac evaluation and/or monitoring is therefore not recommended. In cases of suspected carcinoid syndrome, tumor production of serotonin should be investigated, either with plasma or urinary 5-HIAA and if elevated, an echocardiogram should be performed. Cardiotoxicity of anti-cancer therapy is increasingly recognized and should be considered in cases of symptoms thought to be of cardiac origin (Finet & Tang 2018).

## Hereditary risk

Genetic risk factors for the development of high-grade NENs have not been well elucidated and a detailed search of the literature failed to reveal any studies addressing this question. Established genetic syndromes associated with NETs such as neurofibromatosis 1 and multiple endocrine neoplasia 1 have rarely reported G3 NENs as part of their syndromes (Bordi *et al.* 1997, Chakrabarti *et al.* 2019). Similarly, an association of germline mutations in homologous recombination repair pathways, for example, *BRCA2* and *PALB2* mutations, with G3 NENs is limited to case reports (Bhatla *et al.* 2016, Herold *et al.* 2018). MMR deficiency, however, is being increasingly reported in G3 NENs. In one study, microsatellite instability (MSI) analysis and IHC for DNA MMR proteins were performed in 89 GI or pancreatic NECs or mixed adeno-NECs. MSI was observed in 11 cases (12.4%). All but two of the MSI cases showed *MLH1* methylation and loss of MLH1 protein suggesting that these were not Lynch syndrome-related tumors (Sahnane *et al.* 2015). Another study identified MSI or MMR deficiency in colorectal G3 NEC in approximately 7% of cases, but no clear association with Lynch syndrome has been established (Olevian *et al.* 2016). Current evidence does not suggest inherited syndromes as a possible etiology for the development of G3 NENs and the panel does not recommend routine genetic evaluation based solely on the diagnosis of a G3 NEN.

## Discussion

High-grade NENs are a rare disease entity for which little prospective data are available. In more recent years, the distinction between well-differentiated G3 NET and poorly differentiated NEC has further complicated the management of this disease. The first step to a successful treatment strategy is a strong pathologic evaluation, allowing for the confident interpretation of tumor grade and differentiation. Advances over the past several years have broadened our diagnostic and treatment capabilities for this disease and include a multidisciplinary approach involving pathology, medical oncology, surgical oncology, nuclear medicine, radiation oncology, and interventional radiology. Given the nuanced management that is often needed for the care of patients with high-grade NENs, it is recommended that patients seek care or at least an opinion at a comprehensive neuroendocrine treatment center. With the lack of prospective data available in this disease, the panel also encourages clinical trial participation whenever possible.

## Declaration of interest

The authors declare that there is no conflict of interest that could be perceived as prejudicing the impartiality of the research reported.

## Funding

This work did not receive any specific grant from any funding agency in the public, commercial, or not-for-profit sector.

## Author contribution statement

JRE: consulting—Advanced Accelerator Applications, Ipsen, Lexicon; employment (spouse)—Bristol Myers Squibb, Janssen; research support—Hutchmed, Seagen, Oncolys, Medimmune, Xencor, AstraZeneca, Genentech; stock (spouse)—Bristol Myers Squibb. TRH: consulting—Advanced Accelerator Applications, TerSera, Ipsen, Crinetics, Terumo, Viewpoint Molecular Imaging, ITM Isotopen Technologien Muenchen; research support—Advanced Accelerator Applications, Turnstone Biologics, Basilea, Thermo Fisher Scientific, Agios. TA: consulting—Ipsen, Eisai, Bristol Myers Squibb, Taiho, Amgen, Novartis, Bayer; speaker—Ipsen, Amgen, Novartis. AMB: no disclosures. EB: research support—Merck. AD: consulting—Novartis, Ipsen, Voluntis, Abbvie, Crinetics, Hutchmed, Personalis; research support—Novartis, Eisai, Ipsen, Hutchmed, Guardant Health, Natera. GEH: consulting—Novartis, Canon Medical Systems, Terumo, Boston Scientific, Bayer, Curium Pharma. MF: consulting—Stryker, Seagen; research support—AkesoBio, GlaxoSmithKline, AstraZeneca. JEM: honoraria—Varian; research support—Varian Medical Systems. EM: consulting—Curium, Ipsen, ITM, Telix, TerSera, Lantheus; research support—Curium, ITM, Novartis, Nordic Nanovector. SM: honoraria—Advanced Accelerator Applications/Novartis, Tersera; research support—Advanced Accelerator Applications/Novartis, Ipsen. EN: no disclosures. NR: Ipsen, HRA Pharma, Advanced Accelerator Applications/Novartis; research support—Novartis, Xencor, Corcept Therapeutics, ITM Isotope Technologies Munich. HS: consulting—Helsinn, Excelixis, AstraZeneca, Pfizer, TerSera, Ipsen, Novartis. BU: no disclosures. NV: consulting—ITM, Novartis, AstraZeneca/Daiichi Sankyo, Taiho Oncology, Tersera Therapeutics; honoraria—Guidepoint Global; research support—Zymeworks, Puma Biotechnology. JAC: consulting—Advanced Accelerator Applications/Novartis, TerSera; honoraria—Advanced Accelerator Applications/Novartis, Ipsen, Bayer (spouse), Pfizer (spouse); stock—Merck; research support—Lilly, Sanofi.
